# Cognitive Control

**DOI:** 10.1146/annurev-psych-022024-103901

**Published:** 2024-12-03

**Authors:** David Badre

**Affiliations:** Department of Cognitive and Psychological Sciences, and Carney Institute for Brain Science, Brown University, Providence, Rhode Island, USA

**Keywords:** cognitive control, executive function, cognitive representation, behavioral flexibility, performance monitoring, learning, generalization

## Abstract

Humans and other primates have a remarkable ability to perform a wide range of tasks and behaviors, even novel ones, in order to achieve their goals. Further, they are able to shift flexibly among these behaviors as the contexts demand. Cognitive control is the function at the base of this remarkable behavioral generativity and flexibility. The present review provides a survey of current research on cognitive control focusing on two of its primary features within a control systems framework: (*a*) the ability to select new behaviors based on context and (*b*) the ability to monitor ongoing behavior and adjust accordingly. Throughout, the review places an emphasis on how differences in the content and structure of task representations affect these core features of cognitive control.

## 1. INTRODUCTION

Human behavior is marked by both its generativity and its flexibility. We have a remarkable ability to conduct new tasks to meet our goals, to work toward underspecified or open-ended objectives, and to adapt our behaviors as our contexts and goals change. We can manage multiple goals at once, over multiple timescales, and we can correct course through interruptions, dead ends, and errors. We can not only plan and execute actions that achieve our ends but also withhold action, even when habit or desire might urge us otherwise.

Cognitive control is the mental function at the basis of these abilities. Broadly, cognitive control refers to a class of mechanisms that allow us to organize and execute our thought and behavior in line with our goals (Badre 2020b, Duncan 2013, Egner 2023, Logan 2017, Miller & Cohen 2001, Norman & Shallice 1986). Accordingly, deficits in cognitive control across a wide range of neurological and psychiatric disorders are associated with a loss of adaptive, goal-directed behavior (Devinsky & D’Esposito 2004, Stuss & Benson 1987). Changes in cognitive control over the course of our lifespan shape our ability to live independently and influence a range of life outcomes (Amso et al. 2014, 2019; Blair & Razza 2007; Lacreuse et al. 2020; Luna 2009; Moffitt et al. 2011; Munakata et al. 2012; Reuter-Lorenz & Park 2023). And, despite remarkable gains in recent years, even the most advanced artificial intelligence systems have yet to capture the capacity for computationally efficient behavioral flexibility enabled by human cognitive control (Russin et al. 2020). Thus, cognitive control is central to the course and character of human experience, and, accordingly, understanding cognitive control function has far-reaching implications in psychology.

Despite its importance, cognitive control has been difficult to define and study as a coherent research topic. Indeed, the terms “cognitive control” and “executive function” are used mostly interchangeably and refer to much the same kinds of behaviors. However, they do differ in sense. Whereas executive function tends to emphasize a set of executive skills and abilities, like inhibition or set shifting, cognitive control emphasizes concepts and mechanisms from a control systems theory framework (Badre 2020a, Betzel et al. 2016, Holroyd 2024, Ritz et al. 2022). Accordingly, this will be the framing of this review.

In general, a system is controllable to the degree that it can attain any system state given the right controlling input. Applied to cognition and behavior, goal states can be defined in terms of some real-world outcome or behavior, such as eating a sandwich, taking one’s medicines, or even naming the font color of a word. Thus, control mechanisms are those required for efficient and reliable transition to these behavioral goal states regardless of the initial state and in a stable way across the variability in our world and any intermediate states.

This definition clarifies a common point of confusion in the widely cited dichotomy between automatic and controlled behavior (Norman & Shallice 1986, Shiffrin & Schneider 1984). Automatic behavior refers to rapid, obligatory action patterns that occur in response to a triggering stimulus. Such responses are typically overlearned, effortless, and context independent or habitual. They are contrasted with controlled behaviors that are slow, deliberative, goal directed, and context dependent.

Importantly, however, automatic behavior is still controlled in the control systems sense, in that the triggering stimulus acts as a controlling input to determine a response efficiently and reliably. Indeed, in many cases, precisely this kind of fast automatic control of our behavior is what the situation demands. A baseball hitter or soccer goalie must train rapid responses to a particular visual input of an incoming ball, if they hope to react in time to intercept it with their bat or body. Likewise, a musician sight-reading music or a dancer responding to musical rhythm can do so fluidly because of how they have trained their actions to be controlled by particular bottom-up inputs. These are controlled automatic behaviors because the input is controlling behavior without reference to deliberative decisions or top-down goals. Indeed, this kind of automatic controlled behavior is characteristic of a range of expert behaviors, even ones we do every day, like typing on a keyboard (Logan 2017, 2018).

Of course, we cannot simply rely on environmental stimuli processed through our senses to control our behavior. Indeed, the flexibility of our behavior derives from our ability to make different responses to the same stimulus input as our goals demand, even when stimulus control would dictate another action. In such cases, we can modulate the controlling input using top-down knowledge about goals, rules, norms, or desired outcomes that are derived and maintained by our cognitive system to direct the system to this alternate output state. This context-dependent stimulus-to-response controllability is what is often meant by flexible behavior.

Further, cognitive control is not merely an open-loop system, wherein our top-down input controls the output but is free of information about the state of the output. Rather, we can track our actions, our errors, the amount of mental effort we are expending, or the reward we are receiving. These signals in turn provide feedback to the control mechanism, which adjusts its control input accordingly. In this sense, cognitive control also exhibits properties of a closed-loop system, wherein feedback about the state of the output modulates the controlling input.

Finally, it is important to distinguish between controlling our actions and controlling states of the world itself. We typically do not desire certain action states, like sandwich eating or color naming. Rather, we usually desire future states of the world brought about by those actions, like sating our hunger or succeeding at the task an experimenter assigned us. Thus, the actions we take are those we think likely to cause those desired world states. Likewise, our environment not only provides the input to our senses but also responds and changes as a consequence of our behaviors. Thus, we are control agents in an outer perception-action loop (Fuster 2004) with limited controllability to attain our desired states of the world in finite time through our actions. Nonetheless, we must link our knowledge with our controlled behavior. We must interpret the world, decide what contexts and rules are applicable, make predictions about how our actions will affect the world, and evaluate whether we have achieved the desired outcome. Thus, cognitive control mechanisms interact with knowledge systems that comprehend and model the world in order to bridge the gap between knowledge and action.

These three features of cognitive control—context-dependent stimulus-to-response controllability, monitoring and feedback control, and knowledge–action connection—encompass much of the explanatory scope of cognitive control theory from a control systems perspective. This review focuses on the first two features, which comprise the inner-loop control of action and thought. The outer-loop link between knowledge and action through cognitive control is important, but it is beyond the scope of the present review (see Vaidya & Badre 2022).

A second goal of this review is to emphasize the nature of task representations in cognitive control function. Early influential theories of cognitive control focused on process-based accounts (e.g., Broadbent 1977, Meyer & Kieras 1997, Pashler 1994, Salvucci & Taatgen 2011, Shiffrin & Schneider 1984). Broadly, these models assumed stages of processing, in different schemes, that progress from stimulus to response. Behavioral costs, such as response time (RT), arise from the time spent occupying one or another of these processing stages. Thus, multitasking costs, for example, might be explained in terms of a processing stage that is needed by two or more tasks and acts as a bottleneck (Pashler 1994).

However, recent years have seen a shift in emphasis in favor of representational accounts that consider the information being encoded for a task, its organization, and its transformation. I characterize this as a shift in emphasis rather than as an alternative paradigm, because changes in representation entail effects on the processes that interact with those representations. Nevertheless, as we will see, this shift in emphasis has offered a number of new insights about cognitive control, and it has also opened up new questions that will likely define the field in the coming years.

## 2. CONTEXT-DEPENDENT STIMULUS-RESPONSE CONTROLLABILITY

At their base, cognitive control mechanisms must permit different behaviors to be selected based on internally generated and maintained contextual representations, even when there has been no change in the inputs processed through the senses. As one example, imagine you are conversing with your partner about a surprise gift you plan to give your child, when it occurs to you that the child in question is nearby and might overhear. Perhaps, you pull out your phone and start texting your partner instead, even though they are sitting right in front of you on the couch.

In this example, the environment has not changed. Stimulus control is insufficient to drive your new behavior. However, based on your belief about the situation—that you might be overheard—you start communicating through a different set of actions. Further, while you have certainly texted before, you might never have done so for this particular purpose. Yet, you can easily do so in this novel setting. This ability to make different responses based on the same input in order to meet our goals is an example of context-dependent stimulus-response controllability.

Demanding new responses to stimuli based on a changing context is associated with a replicable set of behavioral phenomena that give clues to the underlying mechanisms of control. For example, a range of tasks—such as Stroop (Stroop 1935), flanker (Eriksen & Eriksen 1974), Simon (Simon & Rudell 1967), Multi-Source Interference Task (MSIT; Bush et al. 2003), and other stimulus-response compatibility manipulations (Kornblum et al. 1990)—variously pit the congruency of contextually appropriate stimulus-response mappings with other, contextually inappropriate stimulus-response mappings. In general, incongruency among these stimulus-response mappings causes worse performance, such as slower RT and increased error, than when they are congruent (Kornblum et al. 1990). In sequential performance when tasks switch, a context change indicates a new task is to be performed using the same stimuli that were used prior to the switch for a different task. This switch likewise results in a cost in RT and accuracy (Monsell 2003).

**Figure 1:**
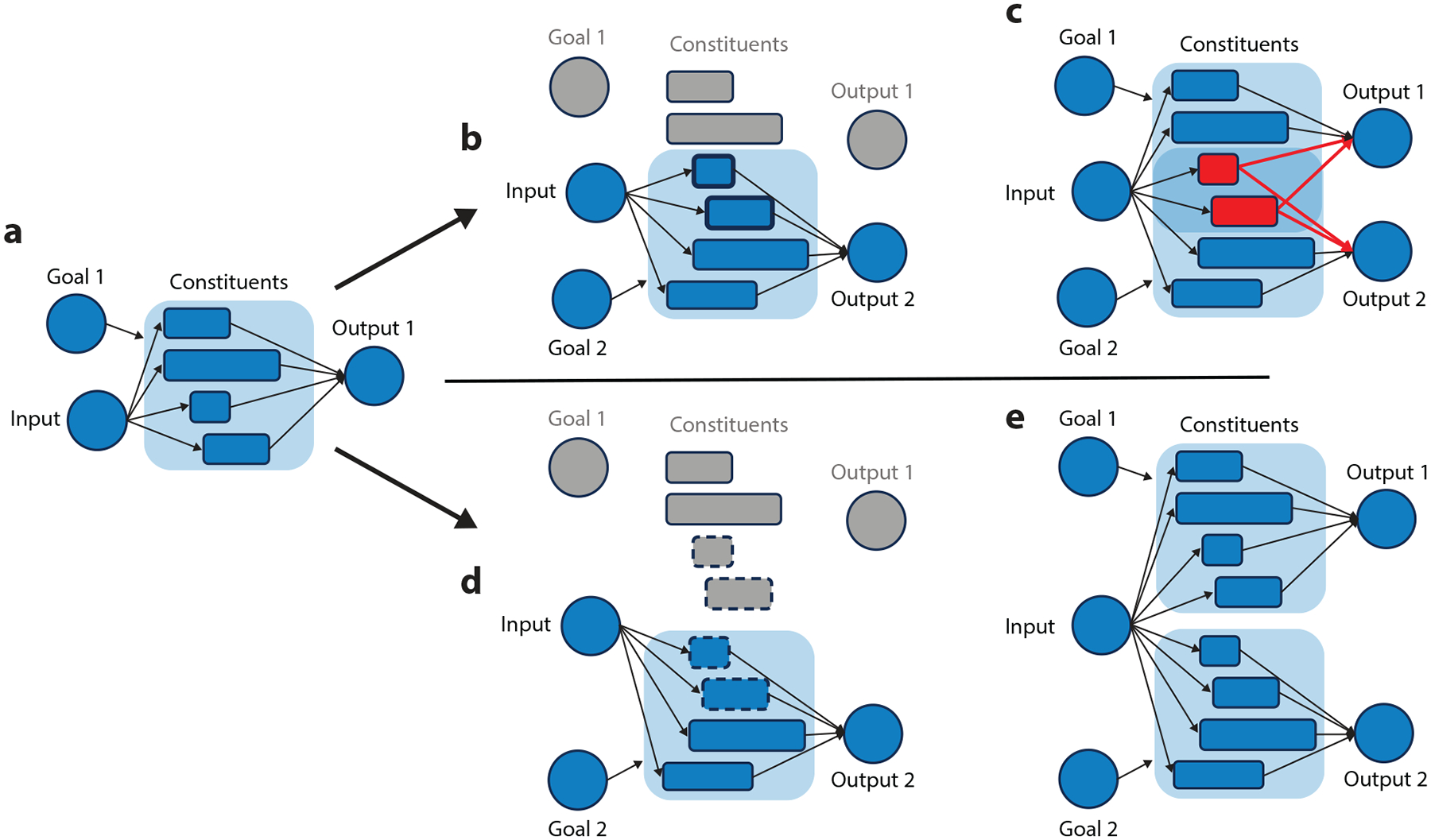
Schematic illustration of the differences between compositional (*b*,*c*) and goal-integrated conjunctive (*d*,*e*) task representational structures. (*a*) This panel represents a task as a progression from input to output. The circles on the left represent external inputs and task goals; the circle on the right represents actions taken. The boxes in the middle represent the various representations that are needed to perform the task, such as categories of stimulus features, concepts retrieved from memory, movement sequences, and so forth. A group of these representations needed for a task is signified by the grouping box. (*b*) Within a compositional structure, a new task (Goal 2) can use shared representations (*weighted border*) to rapidly learn new responses to the same input. (*c*) Activation of both goals, such as during multitasking, produces competition and interference due to the shared representation (marked in *red*). (*d*) Goal-integrated conjunctive representations separate the pathways for each task such that even common constituent representations are unique for the new task pathway (*dashed border*), which can challenge new task learning and generalization. (*e*) The separated pathways can reduce multitasking costs. Behavioral costs in this organization arise when context-integrated pathways are weakly activated.

In recent years, there has been active progress on the mechanisms that solve this stimulus-response controllability problem and explain these costs. Two classes of solution have been proposed that differ based on the assumptions they make about the underlying task representations (Figure 1). Specifically, some solutions assume that context-dependent problems are solved by additive assemblies of compositional task representations. In contrast, other solutions assume that this problem is solved by integrated task representations that are unique to the task being undertaken in a particular context. Each representational solution has its own computational advantages and costs, and accordingly, each has its own control mechanisms and associated behavioral phenomena. We consider these below, in turn.

### 2.1. Compositional Task Representations and the Flexibility Problem

Tasks can be broken into components, such as the stimuli we see, the responses we make, and the rules or policies we rely on to know how to respond given the prevailing inputs (Figure 1a,b). According to one view, these components can be represented as constituents that can be combined and recombined in new ways to generate novel tasks. Loosely adapting a concept from linguistics, tasks represented in this way may be considered compositional in that their function is fully determined by their constituent functions and the organization of those functions. Thus, an everyday task like making coffee can be decomposed into constituents, like the subgoals of measuring grounds or filling a carafe with water, performed in a particular order. Some of these might be reused in other settings, like making tea, in which case they perform the same function but can be organized differently to produce a different overall function.

It follows that the constituents that make up compositional task representations should generalize across contexts and, accordingly, contribute the same subfunction to the tasks of which they are a part. For example, the set of mental representations we need to type on our smartphone might be the same regardless of whether we are texting or emailing or web-browsing. Thus, within a compositional task representation, the individual components of the task, such as the stimuli, goals, responses, and so forth, are context independent.

As is the case with language, compositional task structure can aid us when assembling new tasks for new situations quickly. Using our texting routines to communicate with someone right in front of us without speaking might be a novel use of those routines. However, if we already have the whole apparatus available for the act of texting, then we only need to access those existing representations for this new context. Thus, a benefit of compositional representations is that they provide a ready basis for rapid new learning through the reconfiguration of shared representations, an idea supported in neural network models (Musslick & Cohen 2019). The context-dependence problem might be solved, then, by rearranging shared constituent representations as the context demands.

The fact that people are generative in their behavior (i.e., produce new behaviors and tasks) is often taken as prima facie evidence that humans can represent tasks compositionally. However, to avoid circular reasoning, controlled laboratory studies of learning, generalization, and transfer have tested whether people represent components that generalize across tasks and whether these can support new task learning.

One such line of evidence comes from studies of rule learning within a reinforcement learning setting, which show that people learn and use hierarchical structures to organize task constituents and aid in task learning (Badre & Frank 2012; Badre et al. 2010; Bhandari & Badre 2018; Collins & Frank 2013, 2016; Eckstein & Collins 2020; Eichenbaum et al. 2020; Lee et al. 2022; Li et al. 2022; Tomov et al. 2020; Yanaoka et al. 2024). For example, people can learn to treat one stimulus dimension as a context that determines the policies that relate other dimensions of their environment to their behavior, according to a branching rule structure. People can quickly discover this latent hierarchical structure through reinforcement and will generalize it across stimulus-response mappings. As a consequence, they reach high levels of performance more rapidly compared to a case in which such a structure is not available to be learned and each stimulus-response link must be learned individually (e.g., Badre et al. 2010).

People will even learn a hierarchical structure when that structure confers no particular behavioral advantage over other nonhierarchical ways to represent the task (Collins & Frank 2013). This suggests that people are inclined to represent tasks in a hierarchical way, perhaps because of what a hierarchy affords in terms of compositional representation. Indeed, people can use their hierarchical task representation to support transfer to new tasks, as in the case of rules that share the overall branching structure but with different constituent stimulus-response mappings (Bhandari & Badre 2018, Collins & Frank 2013, Eichenbaum et al. 2020, Yanaoka et al. 2024).

There is also evidence that people can extract constituent representations that recur across different tasks (Franklin & Frank 2020, Liu & Frank 2022, Menghi et al. 2021, Tomov et al. 2021). For example, a recent experiment showed that, in a map-like environment wherein the popularity of goal locations across tasks was independent of the popularity of response mappings across tasks, people could learn to cluster and generalize these two components (goals and response mappings) separately (Franklin & Frank 2020), in essence discovering two constituents that could be used in new tasks. Another recent study trained neural networks on pairs of learning tasks in order to predict the degree to which the pairs shared a common neural subspace. They found that tasks with shared subspaces in the model were more efficiently learned by human participants (Menghi et al. 2021).

Neuroscience approaches have similarly found evidence of compositional task representations in the brain. One prediction is that compositional representations will be evident in patterns of brain activity that correspond to a task constituent independently of the overall task or context. In other words, the brain should exhibit the same pattern of activity to entering text on your smartphone regardless of whether you are doing so to compose an email or to text your partner.

Rapid instructed task learning (RITL) uses a matrix of fully combinable stimuli, rules, and responses, with each combination constituting a new task (Cole et al. 2011). Both familiar and novel combinations can be used, which allows researchers to study the constituent components across both settings. Human participants are able to perform this task, responding correctly even on the first trial of a new combination. Using multivoxel decoding methods, Cole et al. (2011) found that classifiers trained to decode task components based on patterns of fMRI activation in lateral prefrontal cortex (PFC) could generalize across familiar and novel tasks containing those components. Follow-up work showed that the strength of these task representations related to behavioral performance (Cole et al. 2016). Similar evidence of compositional coding in human PFC has been observed during tasks involving compound rules (Reverberi et al. 2012).

Compositional coding is also evident in nonhuman primates. A recent study in macaques trained three different tasks that shared common components, such as a color category subtask or response subtask (Tafazoli et al. 2024). They found common neural subspaces for each of the constituents that were shared across these tasks, as the monkey discovered the correct rule. The strongest generalization of subspaces was found in the lateral PFC. Thus, there is evidence across species of compositional task representations in the brain, specifically in areas like the PFC that are important for cognitive control.

### 2.2. Working Memory Gating and Control in Compositional Systems

A computational challenge for a system that relies on sharing compositional representations across tasks is that those tasks will compete for those common representations, producing incompatible responses, interference, and performance costs (Figure 1c) (Musslick & Cohen 2021). Returning to our example, if you have ever tried talking to someone while writing a text at the same time, you may have accidentally said a word you were trying to type or vice versa. An account of this multitasking cost is that these tasks share a lexical-semantic pathway linking to both verbal and manual response outputs. Activating one runs the risk of activating the other. This competition among shared components provides an account of the behavioral costs described above. During stimulus-response compatibility procedures, associations with a shared response or other task components cause coactivation and interference. Similarly, during task switching or dual task performance, shared component representations can link to multiple incompatible outputs producing competition and interference. Thus, the very same property that gave us the flexibility to text in our scenario above also makes us vulnerable to multitask interference (Musslick & Cohen 2021).

It follows that a role for cognitive control mechanisms in a compositional system is to resolve the conflict that arises between shared components. Several models propose a biased competition mechanism to resolve this conflict, with working memory at its center (Braver & Cohen 1999, Cohen et al. 1990, Desimone & Duncan 1995, Frank & O’Reilly 2006). Working memory allows internal representations of goals, rules, or other contextual information to be maintained even in the absence of external input. Held in working memory, this context is available to serve as a top-down biasing input that allows the context-appropriate action pathway from stimulus-to-response to win its competition against the inappropriate one.

**Figure 2:**
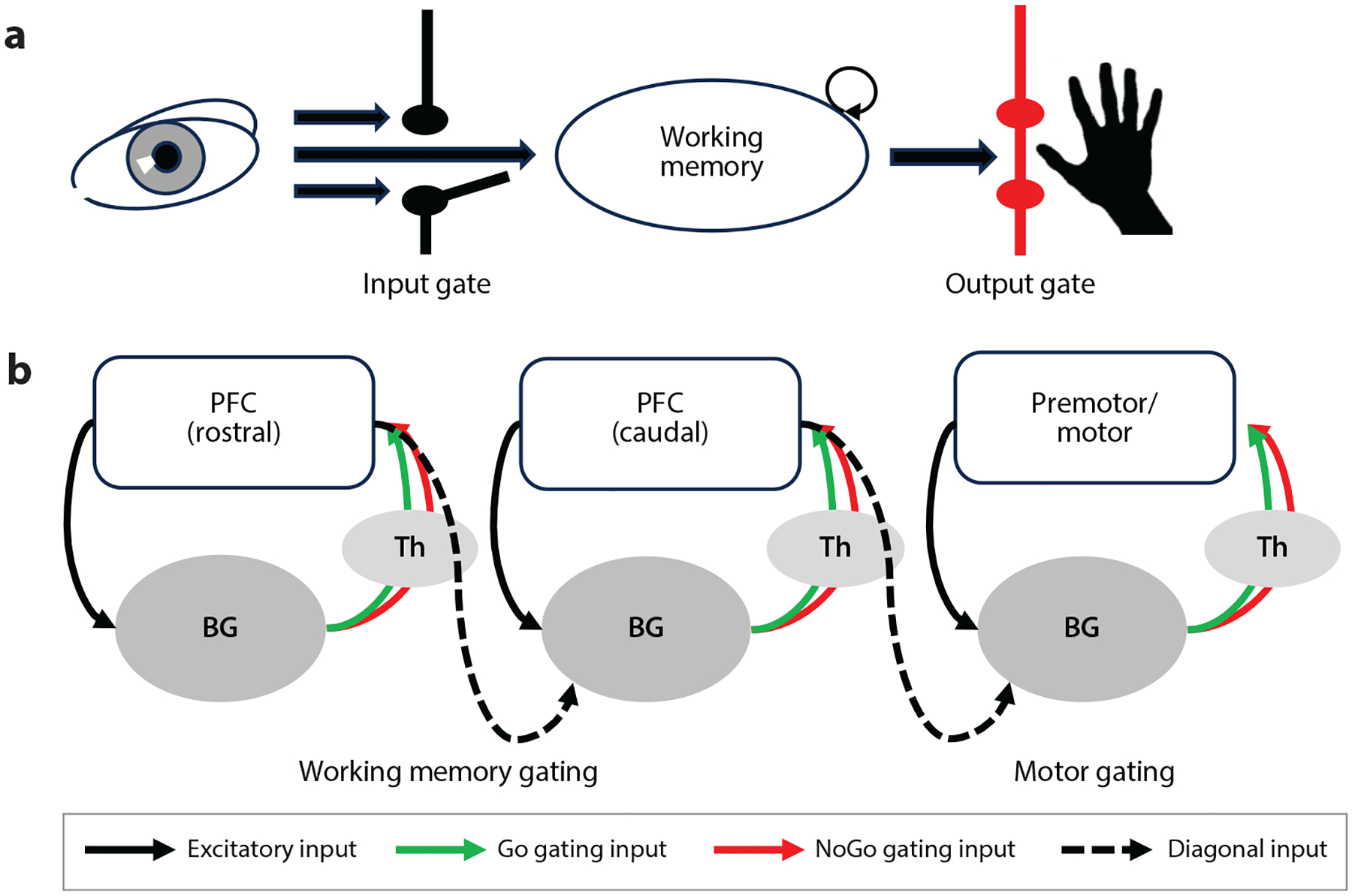
Schematic illustration of working memory gating and its proposed implementation in corticostriatal loops. (*a*) Two gating operations have been proposed. Input gating acts as a maintenance switch, selecting what information from the external world should be maintained in working memory. Output gating selects when and what information from within the working memory can guide action. Panel adapted with permission from Chatham & Badre (2015). (*b*) Schematic of the PBWM model, which proposes that input and output gating are implemented through interacting canonical corticostriatal loops. For each loop, information in the cortex inputs to the basal ganglia (excitatory inputs), which can amplify (Go gating inputs) or suppress (NoGo gating inputs) that cortical representation through the thalamus. Separate loops can gate motor and working memory representations. The output of each loop can influence the other through diagonal connections. A context in the caudal PFC can influence motor gating of the appropriate response, implementing context-dependent response selection. A superordinate context in rostral PFC can influence gating of a subordinate context by the caudal PFC, implementing hierarchical control. Abbreviations: BG, basal ganglia; PBWM, prefrontal cortex, basal-ganglia working memory; PFC, prefrontal cortex; Th, thalamus.

Importantly, not all information is relevant as a contextual input or should be maintained in our capacity-limited working memory. Likewise, multiple contexts could be maintained in working memory at once, but we may want to select when they are deployed as a control signal. The metaphor of a gate has been used to describe this kind of working memory control (Braver & Cohen 1999, Chatham & Badre 2015) (Figure 2a). Specifically, a gated working memory for control is one in which useful contextual information can be selected and placed in a state such that it is accessible at an appropriate later point in time, while distracting information is not (Braver & Cohen 1999, Frank et al. 2001, Hochreiter & Schmidhuber 1997).

It follows that choosing the right gating policy, which specifies what information to gate into and out of working memory for the particular task at hand and when, may be an important determinant of performance, including on new tasks (Bhandari & Badre 2018, Sabah et al. 2021, Unger et al. 2016). For example, Bhandari & Badre (2018) observed that people’s ability to rapidly adapt to changes in task dynamics depended on adopting the correct gating policy, and transferring the wrong gating policy to a new task resulted in inefficient behavior. Importantly, Bhandari & Badre (2018) showed that the effects of gating transfer were independent of changes in the identity of the stimuli or task rules themselves. Thus, the gating policy can operate over different constituent representations to support their efficient incorporation into new tasks, even supporting far transfer (Sabah et al. 2021).

Several mechanistic models of cognitive control incorporate working memory gating, using different implementations (Barak & Tsodyks 2014, Conde-Sousa & Aguiar 2013, Dipoppa & Gutkin 2013, Edin et al. 2009, Frank et al. 2001, Mante et al. 2013, Murray et al. 2017, Santos et al. 2012, Strock et al. 2020, Wang et al. 2004, Zhu et al. 2020). One illustrative example is the prefrontal cortex, basal-ganglia working memory (PBWM) model (Frank & O’Reilly 2006). PBWM comes from a class of models that are compositional in nature, in that they break inputs into component representations that are stable across tasks and contexts (e.g., Rougier et al. 2005).

PBWM implements working memory gating policies through interactions between the PFC, basal ganglia, and thalamus (Figure 2b) that are learned and modulated by dopamine signals that carry information about predicted value. The corticostriatal-thalamic gating circuit in PBWM is canonical, in that the computation for gating working memory shares computational features with the disinhibitory circuit used for movement selection (Albin et al. 1995, Alexander et al. 1986, Graybiel 2008, Mink 1996). That disinhibition acts as a gate, amplifying an adaptive action plan being maintained in cortex. The same circuit can also suppress that motor plan if it is maladaptive. Motor gating serves an adaptive function, allowing the cortex to maintain and consider possible actions without actually performing them (Chatham & Badre 2015).

PBWM assumes that the same computational mechanism supports working memory gating in parallel corticostriatal circuits (Frank & Badre 2012, Frank & O’Reilly 2006). Thus, competing constituent context information maintained in the PFC can be selected using the same disinhibitory mechanism in a different basal ganglia circuit. This provides a mechanism for context-dependent control on the basis of compositional codes held in the PFC.

Elaboration of these circuits also provides a mechanism for enacting hierarchical control within a compositional task framework (Frank & Badre 2012). PBWM assumes that interaction of multiple, parallel corticostriatal gating loops allows superordinate contexts gated out of one loop to influence subordinate contextual gating by other loops. This architecture can capture both the behavioral and neural effects observed during tasks of hierarchical control (Badre & Frank 2012, Frank & Badre 2012, Rac-Lubashevsky & Frank 2021, Ranti et al. 2015).

Multiple convergent lines of evidence support the base features of the corticostriatal gating model (Badre & Frank 2012; Cools et al. 2007, 2009; Frank & O’Reilly 2006; Lee et al. 2015; Moustafa et al. 2008; Stollstorff et al. 2010; Tai et al. 2012). The basic hypothesis that the basal ganglia may be involved in working memory gating has gained support from neuroimaging studies (Chatham et al. 2014, McNab & Klingberg 2008) and studies of patients (Baier et al. 2010). Evidence likewise supports PBWM’s assumptions about the modulation of gating circuits by dopamine (Cools et al. 2007, 2009; Frank & O’Reilly 2006; Moustafa et al. 2008).

Finally, PBWM’s reliance on multiple parallel corticostriatal loops is consistent with evidence of a rostrocaudal organization of the PFC (Abdallah et al. 2022, Badre 2024, Badre & Nee 2018). Evidence from fMRI, patient, and transcranial magnetic stimulation studies in humans suggests that more rostral portions of the PFC are associated with more abstract forms of cognitive control (Azuar et al. 2014; Badre & D’Esposito 2007; Badre et al. 2009; Koechlin et al. 2003; Nee & D’Esposito 2016, 2017). In a review of this literature, Badre & Nee (2018) described this gradient as reflecting sensory-motor, contextual, and schematic control, progressing from premotor to dorsolateral to rostrolateral PFC, respectively. Connectivity between the PFC and striatum follows a similar rostrocaudal organization (Choi et al. 2012, Haber et al. 2020, Verstynen et al. 2012). Moreover, there is some evidence that gating at specific levels of contingency is associated with local corticostriatal activity along this gradient (Badre & Frank 2012, Jeon et al. 2014). Thus, parallel gating loops could control the influence of control representations maintained at different parts of this gradient, consistent with the PBWM account.

In summary, compositional task representations offer a way to naturally accommodate new tasks by breaking them into components and recombining them as the task demands. However, this learning efficiency comes with susceptibility to interference and multitasking costs that must be overcome by control systems. Gating of working memory, such as through corticostriatal interactions, may be a central mechanism by which these conflicts can be resolved in a context-dependent manner.

### 2.3. Context-Integrated Representations and the Flexibility Problem

Returning to our example of texting our partner so that we are not overheard, a second solution to this response selection problem is to generate a unique task representation that integrates our goal with the particular task we are doing (Figure 1d). Thus, rather than relying on selecting the right set of shared compositional representations, we build a new representation of texting that is unique to this situation and distinct from other pathways that incorporate some but not all task features. Thus, when my context changes from talking to my partner to texting, I need only access the integrated task representation I have built for this particular goal-action combination.

An advantage of this integrated task representation is that there is minimal competition from component sharing (Figure 1e). This pathway is distinguished or separated from the pathways I have for other goals or contexts involving texting or communicating. As a consequence, I am less susceptible to competition and interference while relying on this integrated representation, and I should be able to more easily perform this task concurrently with other goals I might have. However, such representations are harder to use when generalizing to new tasks.

Thus, context-integrated task representations comprise the other end of a computational trade-off with compositional task representations (Musslick & Cohen 2021). Specifically, compositional task representations support faster task learning, but they are subject to competition, interference, and multitasking costs. On the other hand, context-integrated representations are harder to learn and generalize, but they can avoid the behavioral costs arising from competition.

This basic computational trade-off can be demonstrated in simulations with feedforward neural networks (Musslick & Cohen 2019, 2021). Networks relying on shared representations are faster to acquire a new task, but they are also susceptible to multitasking costs. Conversely, training networks to build unique goal-integrated action pathways takes longer, but they can multitask more readily.

**Figure 3:**
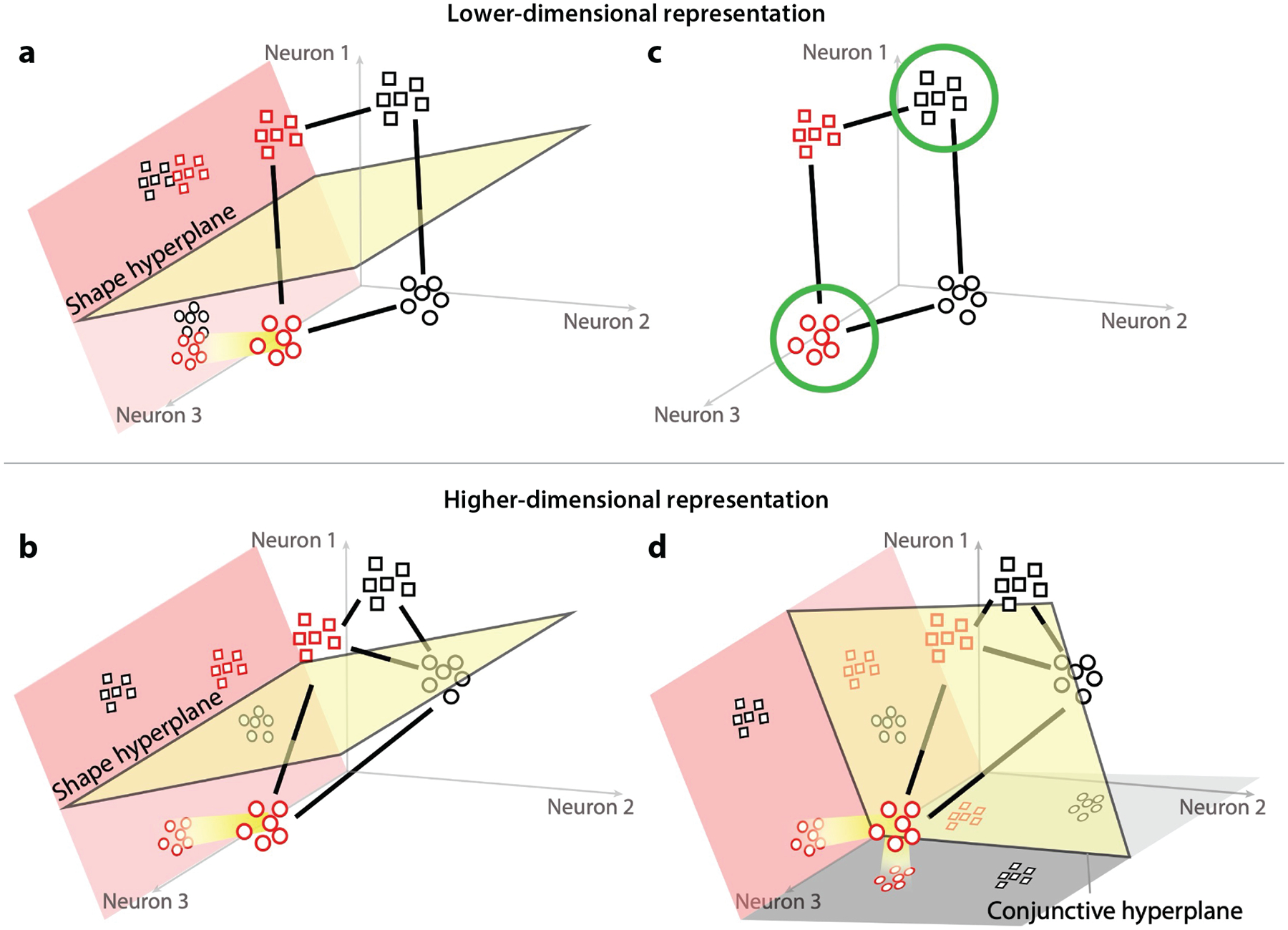
Schematic illustration of the trade-off between generalizability and separability between low- and high-dimensional neural representations (adapted from Badre et al. 2021). Plotted are the firing rates in two populations (*top* versus *bottom row*) of three neurons to inputs differing in shape (*square* or *circle*) and color (*red* or *black*). Distance in space can be interpreted as similarity of activity patterns. The top row shows a lower-dimensional representation in which the variance in activity patterns across the inputs makes a plane, so it can be described by two dimensions. The bottom row shows a higher-dimensional representation wherein the variance in pattern forms a polygon described by three dimensions. Panels *a* and *b* show how each population classifies activity based on shape. In each case, there is a subspace (*pink plane*) where circle versus square can be separated by a linear hyperplane (*yellow plane*) representing readouts from downstream neurons. (*a*) Within the shape subspace, the lower-dimensional representation encodes different colored shapes similarly, abstracting over this irrelevant dimension, which aids generalization and robustness to noise. (*b*) By contrast, the higher-dimensional representation separates these representations based on this irrelevant dimension, making this population more sensitive to noise. Panels *c* and *d* show the readout of a conjunction, for example, wherein a red circle or black square indicates one response and a black circle or red square indicates another. (*c*) There is no hyperplane that divides these classes in the lower-dimensional population. (*d*) By contrast, there is a conjunctive hyperplane in the higher-dimensional population. Higher separability in the high-dimensional representation makes it more expressive and able to separate classes based on small changes in the input.

A related trade-off arises in computational neuroscience, as captured by the state space of recurrent neural networks (Badre et al. 2021, Fusi et al. 2016) (Figure 3). Neural populations receive information about task stimuli, goals, response plans, and so forth and will respond to these inputs with different patterns of activity. Other populations of neurons can then read out information from this population by dividing those activity patterns into separable classes.

Importantly, if a population treats different combinations of inputs as linearly decomposable into a small set of generalized component patterns—i.e., a compositional or low-dimensional representation of the input—then that population will tend to show good generalization of new inputs to those component patterns and can be robust to noise. However, this population will be limited in terms of what mixtures of inputs can be read out at once and may have to rely on serial readout for some combinations, akin to the limitations of compositional task representations.

Conversely, if the population encodes each combination of inputs as a unique pattern of activity—i.e., an integrated or high-dimensional representation of the input—this aids the readout of those combinations as distinct, even if they are distinguished by only a small change in the input. Thus, if the context is the only input feature to distinguish two action conditions, this high-dimensional task representation can readily distinguish these cases as separate, avoiding competition and facilitating their readout. However, this greater separability and expressivity come at the cost of greater sensitivity to noise and of a failure to treat close inputs similarly, which impedes generalization (Badre et al. 2021, Fusi et al. 2016).

Thus, the computational trade-off between low- and high-dimensional neural representations echoes that of compositional versus integrated task representations. As we discuss below, these parallel trade-offs may be important for understanding how the brain represents tasks for cognitive control. Further, this discussion highlights that there are computational benefits to integrated noncompositional representations, and indeed, control systems may take advantage of those benefits.

### 2.4. Evidence for Conjunctive Task Representations During Stimulus-Response Control

There is both behavioral and neural evidence that people rely on goal-integrated representations in the moment-to-moment control of their behaviors (Hommel 2019, Hommel et al. 2001, Logan & Bundesen 2004, Mayr & Bryck 2005, Schumacher & Hazeltine 2016). In general, these representations, sometimes termed event or task files, are conceptualized as unique conjunctions of stimuli, responses, goals, and other task-related features that comprise a particular task event, and their retrieval is tied to efficient action execution. Crucially, these theoretical ideas move beyond classic notions of integration that stopped at stimulus-response bindings (Broadbent 1977, Kornblum et al. 1990, Pashler 1994). Rather, they highlight the importance of integrating context with stimulus and response in order to solve the context-dependent controllability problem. We refer to these context-integrated representations as conjunctive representations (Kikumoto & Mayr 2020).

Broadly, behavioral evidence for conjunctive task coding has come from manipulations in which changing component task features results in nonadditive effects on behavior, an interaction that is inconsistent with the existence of a compositional code across these manipulations. For example, Mayr & Bryck (2005) used a rule-switching task in which individual stimulus-response mappings could be the same while the overall task rule changed. This allowed a test of switching effects at the level of the task rule independently from switching the stimulus, response, and stimulus-response mapping constituents.

A strong compositional view might predict that switching the rule while leaving the stimulus-response mapping the same would benefit behavior compared to a case in which all components change, because one has primed the component stimulus-response representation. However, this was not what was observed. Rather, switching costs on behavior were larger when only one component, such as the rule, switched rather than all of them.

This partial overlap switching effect has been widely observed and replicated in different experimental settings. Specifically, repeating either all features of a task or no features of a task yields faster responses than switching only one feature of the task. From the perspective of the conjunctive code, this effect is due to facilitated access to the full integrated task representation in the first two cases. By contrast, switching only one component can result in multiple conjunctive codes being active, which causes interference and behavioral costs.

Similar partial overlap effects have been observed in other manipulations beyond task switching, including stimulus-response compatibility and sequential compatibility effects (Hazeltine et al. 2011, Hommel 2019). The benefits of practice through repetition also seem to be at the level of the task conjunction, such that high-frequency conjunctions of task conditions will be faster than conjunctions of task conditions encountered less frequently together, even if the lower-level components, such as the individual stimulus-response mappings, have been practiced frequently (Mayr & Bryck 2005).

Neuroscience also provides strong evidence for the hypothesis that people rely on conjunctive task representations for their trial-to-trial performance. First, while we reviewed evidence of compositional coding in the PFC, there is also evidence for context-sensitive representations in this region. Studies using fMRI decoding in the human brain have found that decoding of lower-order features can be sensitive to context, so cross-context decoding often fails in the PFC (e.g., Jung & Walther 2021). Likewise, detailed electrophysiological recordings of neural populations in nonhuman primate PFC find evidence of nonlinear mixed selectivity in cell firing (Rigotti et al. 2013). This complex coding is directly related to high-dimensional task representations that integrate multiple task features into separated coding patterns, and this dimensionality reduces when the monkey makes an error.

In humans, strong evidence for conjunctive coding has come from studies using electroencephalography (EEG). Kikumoto & Mayr (2020) tested participants on a variant of the context-dependent response selection procedure used by Mayr & Bryck (2005). Applying a decoding-based pattern similarity analysis to the EEG data, they were able to track in time the encoding of context-independent stimulus, rule, and response constituent representations as well as the trial-unique conjunction of those three features. They not only found evidence for a conjunctive representation that accounted for variance above and beyond that of the compositional constituents but also found that the strength of the conjunction was among the strongest determinants of trial-to-trial RT. These observations appear highly robust, having been replicated by this group and others (Kikumoto & Mayr 2020, Kikumoto et al. 2022a, Rangel et al. 2023).

Importantly, we can also connect these conjunctive codes to the geometry of neural representations, as introduced above, and their dynamics, which places them in the context of the computational trade-off between generalizability and separability (Kikumoto et al. 2024a). In particular, Kikumoto et al. (2024a) adapted the task used by Kikumoto & Mayr (2020) to incorporate a speed-accuracy trade-off procedure that tested the state of the neural representation when people are able to make correct responses. Using this procedure, they observed that a strong conjunctive code was encoded within a temporally stable and high-dimensional geometry just prior to successful response selection, and this geometry supported efficient controlled behavior. A reason that encoding a conjunction within a high-dimensional geometry may be beneficial is that it favors separability, reducing interference while aiding readout. It follows that the factors that determine whether and when one reaches this stable and separated state may be important determinants of efficient performance.

### 2.5. Summary: Task Representations and Stimulus-Response Controllability

The structure of task representations, such as whether they are compositional or goal integrated, governs an important computational trade-off between generalization and separability. However, this computational trade-off does not mean there is a strict dichotomy in the way cognitive control operates. As we have seen, there is evidence for both compositional and conjunctive representations in the brain. Indeed, it should not be missed that Kikumoto & Mayr (2020) found EEG evidence not only for conjunctive representations but also for simpler context-independent compositional codes alongside those integrated representations. Thus, it is likely that the brain encodes both types of representations, and controlled behavior may depend on their interaction over the course of learning and execution.

Further, while we have pointed out how different theoretical approaches emphasize one or the other structure of representation, no theory makes a strong exclusive claim. PBWM provides a detailed account of how gating mechanisms could be used to manage compositional representations to solve control problems. Nevertheless, the model itself includes integrated representations in its architecture, such as in its hidden layer that multiplexes input dimensions for updating to working memory. Theories that emphasize integrated conjunctive task codes, like Hommel’s (2019) theory of event coding, have been concerned with processes that lead to the activation of the appropriate conjunctive codes, such as through retrieval or binding processes. Nevertheless, these theories also assume that compositional constituent representations can act as cues or associates of those integrated representations.

Thus, this review does not intend to frame a debate about whether cognitive control utilizes one or the other structure to represent tasks. Rather, this review calls attention to the importance of the assumptions a theory makes about representational structure and, in turn, of the computational constraints that structure places on controlled behavior.

One domain where this representational perspective may prove important is in the transition from cognitive controlled to automatic controlled behavior (Kikumoto et al. 2024b). According to one view, automaticity may reflect a transition of control to efficient activation and readout of integrated conjunctive codes. Early on, when transfer and generalization are important, behavior may be more controlled by management of compositional codes that produce positive outcomes but may do so inefficiently, such as through serial decisions about various components with limited multitasking. However, with repeated trials of performance, stronger conjunctive codes control behavior, either due to plastic changes in neural representations or because of easier retrieval of event-like memory trace (Logan 1988). Thus, we may reconceptualize automaticity as facilitated access to task-tailored conjunctive codes—i.e., representations that bind only the task-relevant information needed for a behavioral event into an integrated object.

In summary, it is likely that the task representations change their structure over the course of learning and experience. Thus, the structure of the task will result in an increasingly task-tailored representation that balances the trade-off between generalizability and separability (Bhandari et al. 2024, Flesch et al. 2022). As a consequence, theories of control should take into account that task representations may change their organization over the course of experience.

## 3. MONITORING AND FEEDBACK CONTROL

Sometimes things do not go well. We might make a response we did not intend, or we make the response we intended but do not obtain the outcome we sought. We sometimes catch these deviations and adjust our behavior accordingly. For example, in his classic review of action slips and errors, Norman (1981) describes a subject who stopped themselves just as they were about to pour their tea into an open tomato can (Norman 1981). In this example, the subject detected their error. Of course, it is also a feature of errors that we do not always catch them. Indeed, another of Norman’s (1981) subjects poured juice into their coffee mug but did not notice until the entire cup was consumed, and then only because they saw the thin rim of juice remaining at the bottom of the mug.

Observations like these have led to the inferences that (*a*) there must be a system for monitoring our behavior and its outcomes that provides signals for behavioral adjustment, and (*b*) the monitoring system is separate, at some level, from the system that forms intentions and executes behaviors. Feedback-based cognitive control, then, emerges from the interaction of these systems.

A fruitful theoretical framing for monitoring and feedback-based control, which follows from the control systems view, has been in terms of an optimization problem (Eppinger et al. 2021). In this framing, the control system is making its adjustments to maximize some objective function, such as earning a reward or minimizing error. These adjustments must confront various computational trade-offs. For example, they may balance stability, as in our ability to stay on task and avoid distraction, versus flexibility, as in our ability to nimbly shift to a new task as the context demands; or they may balance exploration versus exploitation, which concerns whether we behave according to a policy we have been using to some benefit or try out a new one that could be even more beneficial.

It is important to note, however, that the system does not need to confront this optimization problem by tuning a single parameter. Consequently, these trade-offs do not need to appear neatly in opposition in behavior to be meaningful, but they can be solved by a division of labor with different components of a system, each governing half of the dilemma. Working memory gating is precisely such a solution to the stability-flexibility dilemma, dividing the problem between stability through memory and flexibility through the gate (Chatham & Badre 2015, Frank & O’Reilly 2006, Hochreiter & Schmidhuber 1997). So, it is likely that optimization through cognitive control systems is multivariate because of these computational trade-offs. The multivariate nature of control raises its own problems for optimization, like the degrees of freedom problem, which are treated more fully elsewhere (Ritz et al. 2022).

Here, in reviewing monitoring and control, we first ask what signals are being monitored by the control system and what defines them. Then we discuss the mechanisms that have been proposed to make adjustments as a consequence of these monitored signals. Importantly, in line with our focus on representation and its structure, we consider how monitoring signals and mechanisms relate to the underlying task representation being controlled.

### 3.1. What Signals Does the Control System Monitor?

The field has focused on four broad kinds of signals that are monitored in laboratory settings: errors, conflict, contextual states, and outcomes. We consider each and its behavioral adjustments in turn.

Monitoring for errors is usually defined in terms of detecting a mismatch between an intended response, based on the context or goals of the present task, and the response that is actually emitted. In everyday life, these errors often emerge as slips of action, such as pouring tea into a tomato can. In the laboratory, they are usually operationalized as pressing the wrong key in a choice response setting (Laming 1979, Norman 1981, Rabbitt 1966, Reason 1990).

Taking this approach, a long-standing finding is that people will adjust their behavior to be both slower and more accurate in the moments following an error (Danielmeier et al. 2011, Laming 1979, Maier et al. 2011, Rabbitt 1966). Notably, post-error slowing occurs in the absence of feedback. And corrections can be at a speed sufficiently fast that they could not have been programmed after the response was executed (Rabbitt 1966). Therefore, error detection does not require explicit feedback, such as witnessing oneself make a mistake, but can rely on internal meta-cognitive signals.

Detected errors are associated with well-known neurophysiological effects, including a marked negativity detectable with scalp EEG at mid-central electrodes in the period directly following the error, termed error-related negativity (ERN) (Fu et al. 2023, Gehring et al. 1990, Holroyd & Coles 2002). Intracranial recordings in humans and nonhuman primates observe the ERN from medial frontal regions around 100 ms to 130 ms after an error (Emeric et al. 2008, 2010; Fu et al. 2019). In the same time range, single neurons within these middle frontal regions increase their spiking to errors (Fu et al. 2019, Sajad et al. 2019). Thus, neurons in medial prefrontal regions signal when errors are detected.

Theoretical models of the ERN have proposed that this signal reflects the mismatch between planned and executed actions (Fu et al. 2023, Gehring et al. 1993), conflict arising from divergent planned and executed actions (Botvinick et al. 2001), or outcome prediction error (Alexander & Brown 2011). In common, however, these models propose that the control system has rapid access to these error signals, which provide feedback to the control system for adjustments.

People also monitor for conflict during a task, even in the absence of error (Botvinick 2007, Botvinick et al. 2001). As discussed above, control often contends with conflict situations in which more than one course of action could be elicited. Within compositional task structures, shared representations result in conflicts between two tasks that both rely on the shared constituent (Figure 1c). Integrated task structures could yield conflict to the degree that more than one conjunctive task file is retrieved at once (Kikumoto et al. 2022a). Detection of conflict can lead to adjustments in behavior, in the absence of error. RT can increase (Verguts et al. 2011), and, crucially, congruency effects are reduced in trials that follow those featuring conflict (Braem et al. 2012, Egner 2007, Gratton et al. 1992, Ullsperger et al. 2005). This conflict adaptation effect is commonly attributed to upregulation of control in response to high conflict on a preceding trial (Botvinick et al. 2001). Analogous to the ERN, detection of conflict has long been associated with activity in the medial frontal cortex, particularly the anterior cingulate (Botvinick et al. 2004). Thus, whether the signals arise from the same cells or not, the superior and medial frontal cortex is a common source of feedback signals from error and conflict monitoring.

Monitoring our context, both internally and in the environment around us, with regard to our ongoing action is also important. The world is dynamic, circumstances change, an action that was relevant one moment becomes irrelevant the next, or unexpected events arise that require adjustments to behavior. Monitoring context is particularly important to navigate these changes actively and organize our broader behavior. The use of prospective memory or strategies like intention offloading (Gilbert et al. 2023) depends on our ability to monitor our context and act accordingly. Likewise, neuropsychological tests of self-organized task performance that are sensitive to patients with control deficits, such as the multiple errands task or the six-elements test, rely on complex forms of context monitoring in order to track progress on multiple goals at once (Burgess et al. 2006, Dawson et al. 2009, Gouveia et al. 2007).

Behaviorally, context monitoring has been studied in a range of tasks in which participants must monitor for cues that indicate a change in their behavior. Perhaps the most well-studied task in this domain is the stop-signal task in which an initiated response must be stopped when a stop-cue is encountered (Logan & Cowan 1984, Verbruggen et al. 2019). As we discuss below, inhibitory mechanisms within the stop-signal task have been considered not only for stopping movement but also for cognitive actions, and these mechanisms are arguably involved when our control system adjusts to any unexpected changes in state.

Finally, outcome monitoring tracks anticipated and realized outcomes from a task. For example, outcome monitoring requires tracking the value we stand to gain from performing a task and our belief about our likelihood of obtaining it. In this context, we can also monitor for conditions that suggest future gains or losses contingent on our behavior, including incentives. When incentives are positive, people’s performance will improve to be faster, less variable, and more accurate (Braem et al. 2012, Esterman et al. 2017, Frober & Dreisbach 2021, Fromer et al. 2021, Soutschek et al. 2014). Further, congruency effects are diminished, which is interpreted as increased control (Padmala & Pessoa 2011).

Importantly, outcome monitoring is influenced not only by the likely receipt of a reward following a task but also by the value of the process that will be or was needed to get to that outcome. This value is most commonly considered in terms of the mental effort required to perform a task (Cavanagh et al. 2014; Kool & Botvinick 2013; McGuire & Botvinick 2010; Sayali & Badre 2019; Shenhav et al. 2013, 2017). Under most circumstances, people find effort to be a cost and so will discount expected value to the degree that obtaining that value requires more mental effort. Tasks that are experienced as more effortful are avoided when people are given free choice (Kool & Botvinick 2013).

The reasons that people find some tasks to be more effortful than others are a matter of ongoing investigation. Experienced effort has been tied to the presence of control demands like conflict, overall task difficulty, and other factors that tax limited capacity systems (Holroyd 2024, Kool & Botvinick 2013, Sayali & Badre 2021, Sayali et al. 2023). One interpretation of the relationship between control or demands on limited capacity system and mental effort is that effort marks a diminished opportunity to gain value from other tasks while performing the current demanding task, an opportunity cost (Kurzban et al. 2013, Musslick & Cohen 2021, Niv et al. 2007).

Finally, recent work suggests that people’s subjective ratings may be particularly important in output monitoring. In particular, subjective ratings of satisfaction, confidence, and effort better accounted for subjects’ decisions about whether to repeat a task or not than did corresponding objective measures of reward, accuracy, or physical exertion (Corlazzoli et al. 2023). This does not mean that we do not implicitly encode those variables; prominent examples of effort-based avoidance in the absence of awareness exist (Kool & Botvinick 2013). Nonetheless, it indicates that subjective metacognitive signals are central to our predictions about outputs and to the feedback available to control systems from output monitoring.

### 3.2. The Representational Structure of Performance Monitoring

An important set of theoretical questions concerns what information is encoded during monitoring and in what form this information is represented. One such question concerns whether the signals that are used for monitoring are domain general or are shaped for specific tasks. Domain-general performance monitoring signals could be useful for upregulating correspondingly general-purpose control signals, such as attention or drive, that might be needed to improve performance across a range of task settings. Alternatively, task-specific monitoring signals could more rapidly elicit task-tailored adjustments. The ability to not only detect a demand but also inform which of many control parameters to adjust may be helped by specificity. Thus, it is informative for theory to know whether feedback signals are domain general versus task specific.

Evidence from the brain has provided some insights into the ways that performance monitoring signals are structured and what information they encode. A recent study tested three different conflict tasks while recording intracranial EEG in humans (Xiao et al. 2023). The authors reported that while conflict signals were decodable within a task, these signals did not generalize across the three tasks they tested. Thus, congruent and incongruent signals were integrated with task information, tailoring the feedback to the particular task and/or kind of conflict that was being monitored.

Of course, intracranial EEG is an aggregate signal, so it is possible that the mixing is somehow due to this aggregated response. Another recent study recorded directly from neurons in human medial frontal cortex and tested conflict coding at both the individual neuron and population levels (Fu et al. 2022). Across three tasks, the authors tested response compatibility-based conflict, flanker-based conflict, and then both types of conflict combined. They found evidence for conflict- and task-specific neurons but also for neurons that were general to a particular kind of conflict across tasks, as in flanker- or compatibility-based conflict. Further, these conflict representations were compositional in nature. Specifically, though there were separate neural subspaces for each type of conflict, these subspaces were combined linearly when both kinds of conflict were present in a task.

To summarize, it is likely the case that both general and task-specific information is available in performance monitoring signals. There is some generality, but it is at an intermediate level, linked to a source of conflict across tasks. This form of feedback might aid optimization by separately shaping the adjustments to each type of conflict. In the next sections, we consider what mechanisms might support these adjustments.

### 3.3. Mechanisms of Control Adjustment: Parametric Control

When people receive feedback signals about unpredicted errors or conflict, outcome, or state changes, they make adaptive responses to these signals. Effects like conflict adaptation and post-error slowing are examples of the behavioral changes we observe in response to those changes. But what are the mechanisms by which these adjustments are made?

One broad class of adjustments people make to their behavior can be described as parametric control. From this perspective, control systems guide behavior according to a range of parameters, at a given level of description. Tuning those parameters in response to feedback or in anticipation of control demands can optimize performance according to an objective function.

Several examples of these parameters have been proposed in the literature and supported experimentally, such as the parameters governing task flexibility, attentional focus, threshold, drift rate, drive, information seeking, and so forth (Eppinger et al. 2021, Fischer et al. 2018, Held et al. 2024, Leng et al. 2021, Ritz et al. 2022, Suh & Bugg 2021). Further, control parameters may generalize across tasks and contexts. For example, a recent study modeled a general task flexibility parameter in the context of a task switching and an Eriksen flanker task (Robinson & Steyvers 2023). They found that parameters defining flexibility in one task could be swapped with those in the other task to account for individual differences. This suggests that the two tasks rely on a common control process governed by a generalizable, trait-like parameter related to flexibility.

There are several mechanisms by which control parameters could be updated in response to feedback. For instance, one can make incremental changes to control parameters on repeated events of a task that will generalize to our behavior on that task moving forward (Egner 2023). For example, the list-wide proportion congruent effect refers to the reduction in congruency effects observed when people encounter a high relative to a low proportion of incongruent or congruent events within a block of trials (Bugg & Chanani 2011, Bugg & Crump 2012). The related list-wide proportion switch effect refers to reductions in switch costs due to a high proportion of switches within a block (Siqi-Liu & Egner 2020). Both effects can be explained in terms of a statistical learning process that adjusts control parameters incrementally over a block (Egner 2023, Jiang et al. 2014).

Importantly, as each trial in these blocks is distinct, this change must be to a component or process that generalizes across trials. Incremental changes to a gating threshold are one example of such a component, though this has not been tested directly in the case of list-wide effects. Nevertheless, models like PBWM include mechanisms for incremental adjustments to gating based on reinforcement learning that result in independent changes to both stability and flexibility tailored to the control demands of the task (Frank & O’Reilly 2006).

Of course, the incremental learning described above would constitute a kind of proactive control, which anticipates expected control demands (Braver 2012). However, at least with regard to mechanisms like working memory gating, there is evidence these can also act reactively, adjusting these general parameters in response to unexpected changes in control demands. As one example, evidence from a range of sources, including patients undergoing brain stimulation and recording and concurrent EEG-fMRI in healthy individuals, suggests that the basal ganglia gating circuit forms a closed loop controller with the monitoring regions of the medial frontal cortex (Basu et al. 2023, Cavanagh et al. 2011, Frank et al. 2015, Holroyd & Coles 2002, Ratcliff & Frank 2012). Specifically, changes in competition or decision uncertainty during a task drive activity in the medial frontal cortex, which, in turn, leads to basal ganglia dynamics that elicit gate closing. During conflict, this has the consequence of increasing the decision threshold, slowing responding, and allowing more information to accrue. Importantly, these kinds of adjustments are general and not tied to a specific trial or task event type.

However, not all mechanisms for control adjustment are task general. Another class of mechanism for enacting control adjustments extends the integrated task representation model to include control parameters as part of the conjunctive task code (Blais & Bunge 2010, Blais et al. 2007, Dignath et al. 2019, Mayr et al. 2003, Verguts & Notebaert 2009). In this theory, when one performs a trial of a task, one can integrate into a conjunctive representation not only the specific stimulus, response, and context but also the various control parameters that were used. For example, when performing Stroop, one might encounter the word “red” written in a green font. If the task is to name the color, rather than read the word, one would bind not only the color name, font color, task rule, and verbal response but also the control parameters, like the greater attentional focus required. Thus, on subsequent trials, encounters with the word red written in green would elicit that prior control setting. This would allow rapid adjustments to specific task events for which one has encoded a conjunctive representation, though it would not permit generalization to new items per se.

One line of evidence for this retrieval mechanism of parametric control adjustment has come from studies that manipulate the association of particular items with control demands, independently of set- or block-level effects. For example, one can manipulate the likelihood that individual stimuli will be encountered during incongruent trials of a response compatibility experiment (Bugg & Hutchison 2013) or during switch trials of a task-switching experiment (Chiu & Egner 2017). Doing so will reduce congruency effects for those items in the former case and switch costs for those items in the latter case. These item-specific proportion congruency and switching effects occur independently of list-wide congruency or switching effects. A recent integrative theoretical review of this area highlighted the independence of these item-specific versus list-wide effects and raised the possibility that they may arise from different learning mechanisms (Egner 2023).

In the context of this review, the distinct mechanisms of learning and transfer between these forms of control appear consistent with each one operating within a control system that adjusts behavior on the basis of compositional and integrated task representations. When possible, integration of control parameters within a conjunctive code supports rapid adjustment with high specificity to a particular event. However, often one must generalize away from specifics and make adjustments that can affect a particular constituent or class of constituent representations (e.g., all the colors are difficult to distinguish and require more attention). Thus, the control system likely has different mechanisms to address these various optimization problems, supported by different representations.

### 3.4. Mechanisms of Control Adjustment: Inhibitory Control

Inhibitory control is widely recognized as fundamental to cognitive control. However, this concept is sometimes misleading, conflating a description of behavioral change with an underlying process of inhibition (Macleod et al. 2003). Observations of slowed responses or of a failure to emit a response are sometimes ascribed to inhibitory control. However, these changes in behavior can stem from processes that are not fundamentally inhibitory. We can effectively avoid doing one thing by actively choosing another. Likewise, indirect interference or blocking can cause slowing without a direct inhibitory process. Thus, in this review, we define an inhibitory process as one that directly countermands or acts to suppress or restrain an otherwise activated representation that would have driven behavior had we not inhibited it.

Inhibition may be the first, fast adjustment our system makes to changes in context or other prediction errors. Context monitoring often alerts us to situations when an intended or ongoing response is no longer appropriate. In these cases, we might want to stop whatever we are doing to avoid an unwanted outcome. Norman’s (1981) participant not only detected that they were about to pour tea into a tomato can but also stopped themselves from doing so. Inhibition of action in response to a change in context can also make room for resetting and replanning.

In the laboratory, the stop-signal task is widely acknowledged as testing inhibitory control (Verbruggen et al. 2019). This task requires a speeded response to a go stimulus on every trial. The go stimulus is followed by a stop signal on a subset of trials, usually around 25%, requiring the participant to stop themselves before they respond.

Behavior in the stop-signal task is commonly modeled by a race model, which assumes that the go and stop process are independent and race each other for control of behavior (Logan & Cowan 1984, Logan et al. 2014). When the delay between the go and the stop stimulus, or stop signal delay (SSD), is short, people are more successful at stopping themselves than when the delay is long. Under the assumptions of the race model, by setting the SSD so that people are equally likely to fail as to succeed at stopping, the difference between the SSD and the median Go RT provides an estimate of stop-signal RT (SSRT), providing a measure of the efficacy of stopping. While there are several challenges to the race model and its assumptions of independence and process purity (e.g., Bissett et al. 2021), under a standard set of conditions, SSRT can capture variance in inhibitory control within and between subjects.

The neural mechanisms of inhibitory control in the stop-signal task are among the best supported, with data from multiple modalities and causal evidence from both lesion studies and circuit-level work in animal models. The most influential model links inhibition in this task to interactions between the PFC, specifically the right inferior frontal gyrus and the pre–supplementary motor area, and the subthalamic nucleus (STN) (Aron et al. 2016). This pathway, often termed the hyperdirect pathway (Mink 1996), bypasses the striatal gating circuits described above by direct input from PFC to the subthalamic nucleus. Activation of this hyperdirect pathway will cause the rapid cessation of action (Chen et al. 2020).

There has been less work on what information is encoded by representations in the PFC-STN circuit as opposed to its stopping function and dynamics. Nonetheless, there are some clues. There is evidence that the operation of this circuit is not only fast but also broad, resulting in nonselective suppression of motor responding (Wessel & Aron 2017, Wessel et al. 2022). Further, recent evidence suggests that inhibition in the stop-signal task involves two stages (Diesburg & Wessel 2021). An initial pause phase is both fast and nonselective, carried out by the hyperdirect pathway. A following cancel phase involves more complete, selective suppression of an unwanted response (Mosher et al. 2021), possibly supported by the corticostriatal pathway. Thus, pausing can allow slower corticostriatal gating pathways to decide whether to stop a specific action or, in tasks where a new response is needed, to select a new response as needed.

A range of neuroscience evidence, including physiological markers of the activity in this circuit, such as bursting in the beta frequency band (Wessel 2020), indicates that this stopping circuit may also inhibit cognitive representations, not just the motor response (Wessel & Anderson 2024). In light of work showing that the thalamus may be organized according to a low-dimensional representation of cortical networks (Hwang et al. 2022), these observations raise intriguing hypotheses regarding the functional resolution of fast inhibitory circuits through the PFC-STN circuit and their influence on clusters of cortical activity through the thalamus, both motor and nonmotor (Wessel & Anderson 2024).

Finally, while its effect may be nonselective, inhibitory control can operate over both compositional and integrated task representations. Often tasks like stop-signal are assumed to operate on constituent motor responses. However, Kikumoto et al. (2022b) found that the strength of conjunctive codes determined the success of inhibitory control, in that stronger conjunctive representations were more likely to result in stop failures. Moreover, this was not simply due to a global inhibitory effect. The conjunctive and response representations were suppressed on successful stops, but the rule and stimulus representations were not. Thus, another benefit of integrated conjunctive representations is that they can serve as a target of inhibitory control mechanisms to stop a full action plan, without needing to suppress each individual constituent representation.

## 4. CONCLUSIONS

In this review, we have surveyed recent work on two major topics within the control systems framework: context-dependent stimulus-response controllability and performance monitoring and adjustment. A theme throughout has concerned how the structure of task representations—particularly, compositional versus context-integrated conjunctive representations—influences cognitive control. We have seen that both kinds of representations are evident in the brain and influence behavior. It is likely that representational structure changes with experience and is increasingly tailored to balance a trade-off between generalization and separability.

Looking forward, this representational framing raises a number of important new questions for scientists interested in cognitive control. The exact linkage between different representational structures and controlled behavior remains to be defined in specific terms, and consequently, it remains difficult to predict how changes in representational structure affect cognitive control. Cognitive control is known to differ considerably between individuals and over the lifespan. We do not yet understand how these differences relate to individuals’ underlying representational structures. There is also increasing recognition that we must understand control in the context of complex, ecologically valid tasks and settings. It seems likely that the representational claims and mechanisms discussed in this review will be placed under strain when they must account for complex and dynamic tasks outside the laboratory. However, doing so is essential for both fundamental theoretical reasons and for more direct application of our knowledge. Regardless of whether research focus is on fundamental mechanisms, individual differences, mental and brain health, or other topics, cognitive control theory moving forward should be explicit regarding the assumptions that are made about the structure of task representations and the computational constraints they bring.
